# Central Role of Spinal MRI in Diagnosing Medullary Cavernoma Revealed by Hematomyelia: A Case Report and Literature Review

**DOI:** 10.7759/cureus.110171

**Published:** 2026-06-03

**Authors:** Meryem El Bardai, Ismail Chaouche, Hajar Ouazzani, Nizar El Bouardi, Baderddine Alami, Y Lamrani, Meryem Boubbou, Mustapha Maaroufi

**Affiliations:** 1 Department of Radiology, CHU Hassan II, Fez, MAR; 2 Department of Radiology, Hassan II University Hospital, Sidi Mohamed Ben Abdellah University, Fez, MAR; 3 Department of Mother and Child Radiology, Hassan II University Hospital, Sidi Mohamed Ben Abdellah University, Fez, MAR; 4 Department of Mother and Child Radiology and Interventional Imaging, CHU Hassan II, Fez, MAR

**Keywords:** hematomyelia, medullary cavernoma, mri, neurology, neuro-surgery, radiology, vascular malformation

## Abstract

Medullary cavernomas are rare vascular malformations of the central nervous system composed of dilated, fragile vessels prone to spontaneous hemorrhage. Although spinal localization is less common than cerebral involvement, bleeding may result in severe neurological deficits and significant morbidity. We report the case of an 85-year-old patient with no major relevant history who presented to the emergency department with acute, intense cervical spinal pain associated with rapidly progressive tetraparesis. Neurological examination revealed marked weakness of all four limbs. Initial laboratory tests were unremarkable. Spinal magnetic resonance imaging demonstrated a cervical intramedullary cavernoma associated with surrounding hematomyelia and local mass effect, confirming the diagnosis of hemorrhagic medullary cavernoma. The patient was referred for multidisciplinary neurosurgical assessment. This case highlights the importance of considering hemorrhagic medullary cavernoma in elderly patients presenting with acute spinal pain and rapid neurological deterioration, and underscores the value of prompt MRI and individualized multidisciplinary evaluation for early diagnosis and management.

## Introduction

Medullary cavernoma is a rare vascular malformation composed of dilated, fragile vessels that may bleed spontaneously [1,2]. Reported annual hemorrhage rates vary across studies, ranging from approximately 1.4% to 6.8%; the rehemorrhage rate is 2% [3], with a consistently higher risk observed following an initial hemorrhagic episode [4,5]. Hemorrhage may remain confined within the cavernoma or extend into adjacent spinal cord structures, resulting in hematomyelia and acute neurological deterioration [1,2,6].

Clinical presentation is often abrupt, characterized by severe localized spinal pain followed by rapidly progressive neurological deficits, including paraplegia or tetraplegia, sensory disturbances, and sphincter dysfunction. Neurological examination may initially reveal flaccidity and hyporeflexia before evolving toward spasticity, reflecting acute spinal cord injury secondary to hemorrhagic compression and tissue damage [2,7,8].

Spinal MRI remains the gold standard imaging modality for diagnosis, typically demonstrating a heterogeneous intramedullary lesion with peripheral T2 hypointensity related to hemosiderin deposition and recent hemorrhage [6]. However, diagnosis may be challenging, particularly in elderly patients, in whom acute spinal cord symptoms may mimic other vascular, degenerative, inflammatory, or neoplastic conditions [7].

Surgical management is indicated in cases of progressive or severe neurological symptoms, with the goal of maximal safe resection of the cavernoma to reduce the risk of recurrent bleeding. Clinical and radiological monitoring is required in cases of asymptomatic or stable cavernomas [1,6].

We report the case of an 85-year-old woman presenting with acute cervical spinal cord compression secondary to hematomyelia caused by a medullary cavernoma. This case highlights the crucial role of spinal MRI in the diagnostic process and illustrates the diagnostic and therapeutic challenges posed by this rare condition in elderly patients.

## Case presentation

An 85-year-old woman with no significant medical or surgical history was admitted to the emergency department with acute spinal cord compression presenting as cervical pain, tetraplegia, and sensory and sphincter disturbances. No history of trauma was reported.

A cervicothoracolumbar spinal MRI (Figures 1-3) demonstrated an intramedullary cervical lesion exhibiting mild T1 hyperintensity and T2 isointensity, surrounded by a peripheral T2 hypointense rim, with mild enhancement following gadolinium administration. No associated venous vascular malformation was identified. The main differential diagnoses included intramedullary neoplasms such as ependymoma and astrocytoma, as well as inflammatory conditions such as transverse myelitis. However, the presence of intralesional hemorrhagic features, the characteristic hemosiderin rim, and the acute clinical presentation strongly favored a diagnosis of a hemorrhagic intramedullary cavernous malformation. These imaging findings were therefore considered consistent with a hemorrhagic intramedullary cavernoma. The patient was discussed at a multidisciplinary neurosurgical team meeting, where management options were reviewed. A decision regarding the treatment strategy was made based on the clinical context and imaging findings. Long-term clinical and radiological follow-up could not be obtained, as the patient was either lost to follow-up or declined further management after the initial evaluation. 

**Figure 1:**
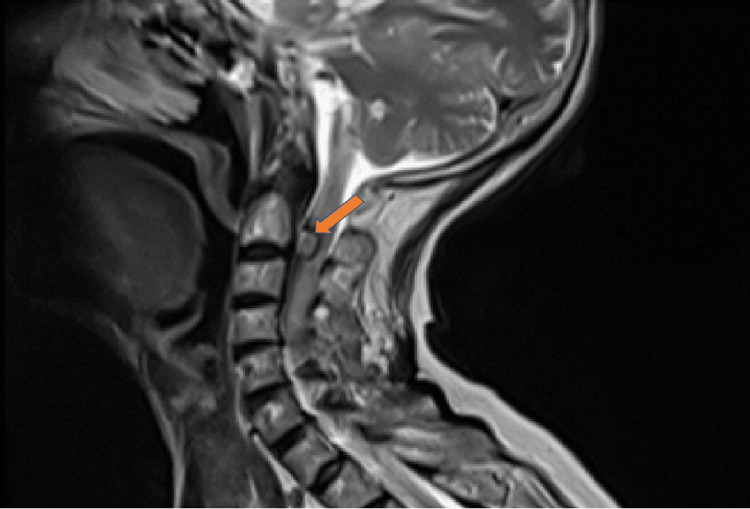
Sagittal T2-weighted MRI image showing an intramedullary oval-shaped lesion with T2 isosignal, surrounded by a hypointense rim characteristic of cavernomas (arrow: cavernoma)

**Figure 2:**
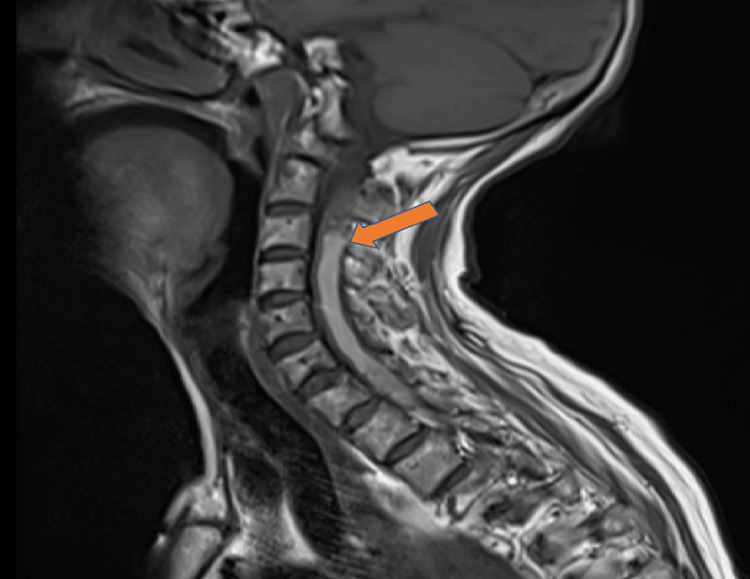
Sagittal T1-weighted MRI image showing the intramedullary T1 hypersignal (hematomyelia)

**Figure 3:**
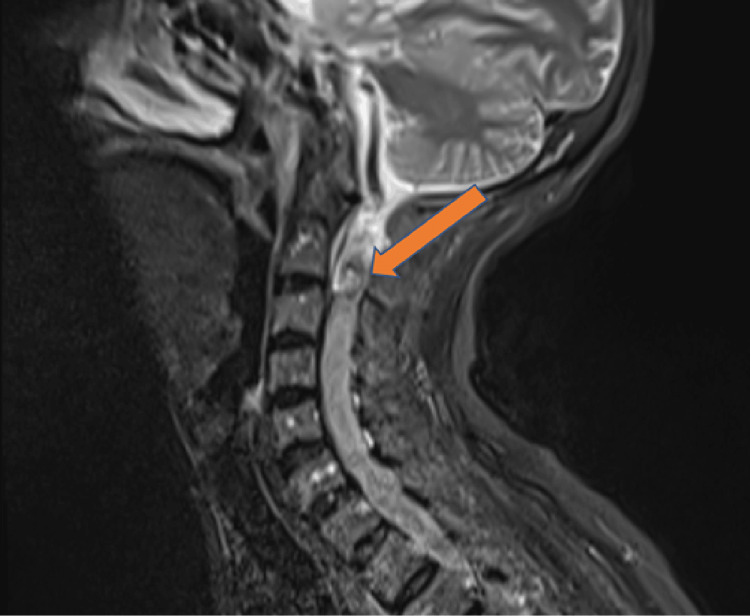
Post-contrast fat-saturation MRI image showing slight enhancement of the spinal cavernoma

## Discussion

Intramedullary spinal cavernomas are rare vascular malformations representing a small proportion of spinal vascular lesions. They consist of clusters of dilated vascular channels without intervening neural tissue and are characterized by low-flow circulation, predisposing them to recurrent micro- or macrohemorrhage. Clinical presentation is highly variable, ranging from incidental findings to progressive myelopathy or acute neurological deterioration secondary to hematomyelia [1,5,8].

In the present case, acute neurological symptoms associated with intramedullary hemorrhage represented a significant diagnostic challenge. Hemorrhagic spinal cord lesions may mimic several aggressive pathologies, including ependymoma, astrocytoma, hemangioblastoma, and hemorrhagic metastasis [3,7]. Acute bleeding can obscure the characteristic imaging appearance of cavernomas and complicate the diagnostic process. Therefore, the diagnosis often relies on a combination of clinical evolution, radiological findings, and exclusion of malignant etiologies [9].

MRI remains the gold standard for the diagnosis of spinal cavernomas. Typical findings include a well-circumscribed intramedullary lesion with heterogeneous signal intensity related to blood products of different ages and a peripheral hypointense rim on T2-weighted images caused by hemosiderin deposition. A characteristic "popcorn-like" appearance may be observed because of mixed signal intensities reflecting recurrent hemorrhages at different stages. Areas of calcification may occasionally be present and contribute to signal heterogeneity. Gradient-echo T2* and susceptibility-weighted imaging (SWI) sequences are particularly sensitive for detecting hemosiderin deposition, microhemorrhages, and calcifications. Angiography is usually negative because of the low-flow nature of the malformation, which also helps differentiate cavernomas from other spinal vascular abnormalities [3,6,8].

Several radiological and clinical characteristics may help distinguish cavernomas from other hemorrhagic intramedullary spinal lesions. Cavernomas are generally well delineated, associated with recurrent hemorrhhagic changes, and show limited surrounding edema. In contrast, neoplastic lesions such as astrocytomas or metastases more commonly demonstrate progressive infiltrative extension and marked spinal cord enlargement. However, overlap may occur, particularly after acute hemorrhage [7,10].

The main radiological, clinical, and pathological features differentiating cavernomas from other hemorrhagic intramedullary spinal lesions are summarized in Table 1 [7,9-12]. 

**Table 1:** Differential diagnosis of hemorrhagic intramedullary spinal lesions MRI: magnetic resonance imaging.

Feature	Cavernoma	Ependymoma	Astrocytoma	Hemorrhagic metastasis
Typical age	Young to middle-aged adults	Adults	Children and young adults	Older adults
Clinical course	Recurrent or acute hemorrhagic episodes	Progressive neurological decline	Progressive neurological decline	Rapid deterioration
Lesion margins	Well circumscribed	Relatively well defined	Infiltrative	Infiltrative
Hemosiderin rim on T2 MRI	Frequent and characteristic	Occasional	Rare	Rare
Contrast enhancement	Mild or heterogeneous	Strong and homogeneous	Variable	Marked enhancement
Surrounding edema	Limited	Moderate	Extensive	Marked
Cord expansion	Mild	Frequent	Frequent	Variable
Associated hemorrhage	Common	Possible	Rare	Common
Angiography	Usually negative	Negative	Negative	Negative
Histopathology	Dilated vascular channels without neural tissue	Glial tumor with ependymal differentiation	Diffuse astrocytic proliferation	Secondary malignant infiltration

Complete microsurgical resection remains the treatment of choice for symptomatic spinal cavernomas, particularly in patients presenting with hematomyelia or progressive neurological deficits. Surgical management aims to prevent recurrent hemorrhage and irreversible spinal cord injury. Previous studies have reported favorable neurological outcomes in most operated patients, especially when treatment is performed before severe chronic myelopathy develops. However, transient postoperative neurological worsening may occur because of manipulation of the spinal cord [1,8].

Conservative management with clinical and radiological follow-up may be considered in asymptomatic or neurologically stable patients. Radiotherapy and radiosurgical techniques remain controversial and are not routinely recommended [1,8].

This case highlights the importance of considering cavernoma in the differential diagnosis of hemorrhagic intramedullary spinal lesions. It also emphasizes the crucial role of MRI in establishing the diagnosis, guiding surgical management, and improving neurological outcomes through early intervention.

Thus, MRI plays a central role in diagnosis, therapeutic decision-making, and follow-up, directly contributing to improved functional prognosis in patients with spinal cavernomas [1,8].

## Conclusions

Although rare, spinal cavernomas can cause hematomyelia, leading to acute neurological deficits and may mimic more aggressive intramedullary lesions. Spinal MRI remains the key examination for rapid and accurate diagnosis, allowing assessment of the extent of hemorrhage, lesion characterization, and surgical planning.

This case highlights the diagnostic challenge of hemorrhagic intramedullary spinal lesions in the acute setting and emphasizes the importance of including cavernoma in the differential diagnosis of spinal cord hemorrhage. It further underlines that careful MRI interpretation, combined with early clinical suspicion, can significantly influence diagnostic orientation and subsequent therapeutic decision-making in patients presenting with hematomyelia.
